# Utilizing Crushed Recycled Marble Stone Powder as a Sustainable Filler in SBS-Modified Asphalt Containing Recycled Tire Rubber

**DOI:** 10.3390/polym17010070

**Published:** 2024-12-30

**Authors:** Byungsik Ohm, Sang Yum Lee, Tri Ho Minh Le

**Affiliations:** 1 Department of Highway & Transportation Research, Korea Institute of Civil Engineering and Building Technology, 283 Goyangdae-Ro, Ilsanseo-Gu, Goyang-si 10223, Gyeonggi-Do, Republic of Korea; bseom@kict.re.kr; 2 Faculty of Civil Engineering, Induk University, 12 Choansan-ro, Nowon-gu, Seoul 01878, Republic of Korea; 3 Faculty of Civil Engineering, Nguyen Tat Thanh University, 300A Nguyen Tat Thanh Street, District 4, Ho Chi Minh City 700000, Vietnam

**Keywords:** crushed recycled marble stone powder, SBS polymer, recycled tire rubber, sustainable asphalt, innovative filler, environmental impact, dynamic modulus

## Abstract

The increasing demand for sustainable construction materials has driven the exploration of alternative fillers in asphalt production. Traditional asphalt mixtures rely heavily on natural aggregates and petroleum-based binders, contributing to environmental degradation. This study proposes an innovative solution by utilizing Crushed Recycled Marble Stone Powder (CRMSP) as a sustainable filler in SBS polymer-modified asphalt containing high volumes of recycled tire rubber, addressing both resource depletion and waste management concerns. A total of 10 asphalt mixes were formulated with varying CRMSP content (0–100% as a replacement for conventional filler) and SBS polymer (3–5%), and their performance was evaluated through Marshall stability, flow, volumetric properties, and dynamic modulus tests. The results demonstrate that incorporating CRMSP up to 75% significantly enhances asphalt’s mechanical properties. The 75% CRMSP mix showed superior stability (19.2 kN, 24.1% improvement), flow (4.6 mm, 4.5% improvement), and resistance to rutting (lowest rut depth: 0.18 mm, 16.7% reduction) compared to the control mixture. Dynamic modulus testing further confirmed the improved resistance to deformation, with the 75% CRMSP mix exhibiting the highest modulus (6.9 GPa, 15.0% improvement). This research highlights the potential of CRMSP as an innovative and eco-friendly alternative filler, improving asphalt performance while reducing environmental impact. By offering a sustainable way to recycle marble waste and tire rubber, this study paves the way for greener, cost-effective asphalt formulations. Future studies should focus on real-world applications, durability, and long-term performance to validate the potential of CRMSP-modified asphalt in commercial use.

## 1. Introduction

Increasing environmental concerns and the need for sustainable infrastructure solutions have led to growing interest in the recycling of materials in the construction industry [1]. Asphalt, a critical material for road construction, traditionally relies on virgin materials, which contribute to resource depletion and environmental pollution [2]. With the global demand for better-performing, more sustainable road surfaces, it is imperative to explore alternatives that do not compromise the quality and durability of roadways [3].

Conventional fillers, such as limestone and sand, have been widely used in asphalt mixtures to improve their mechanical properties [4]. However, the growing emphasis on sustainability and cost-effectiveness has led to the exploration of alternative additives. Several studies have examined the potential of using industrial by-products, waste materials, and natural substances as substitutes for conventional fillers in asphalt mixtures [5]. Recycled materials such as fly ash, slag, and recycled concrete aggregates have been studied for their potential to replace traditional fillers [6]. Research suggests that these alternatives can enhance the performance of asphalt in terms of stiffness, durability, and resistance to moisture damage. For example, a study by Choi et al. (2018) investigated the use of tire-derived fuel fly ash as filler materials [5], showing improved performance. Similarly, the inclusion of molybdenum tailings as fillers has been shown to reduce the environmental footprint of asphalt mixtures while maintaining, or even enhancing, their mechanical properties [7]. However, the effectiveness of these alternative fillers depends on the specific properties of the waste material, its compatibility with asphalt binder, and its impact on the long-term performance of the mixture. In many cases, the integration of alternative fillers can lead to challenges such as variability in material properties, which necessitates further optimization and testing.

Styrene-Butadiene-Styrene (SBS) polymer modification of asphalt binders is a well-established practice in the asphalt industry. Recent studies have explored various approaches to enhancing the performance of SBS-modified asphalt, often incorporating waste tire rubber. Huang et al. [8] found that waste tire rubber combined with SBS improved asphalt’s high and low-temperature performance. However, adding large amounts of tire rubber can cause brittleness and reduce fatigue resistance [9]. Studies like Aliakbari et al. [10] and Yu et al. [11] suggest that alternative fillers, including hybrid systems, can improve both environmental sustainability and performance. SBS-modified asphalt (SBSMA) has gained significant attention due to its enhanced performance characteristics, particularly in terms of improved elasticity, durability, and resistance to high temperatures [12]. The key reason for incorporating SBS polymer into asphalt binder is to enhance the binder’s viscoelastic properties, making it more resistant to deformation and fatigue cracking. Research has shown that SBS modification improves the rutting resistance of asphalt by reducing the binder’s temperature susceptibility [13]. The polymer interacts with the asphalt binder to form a network structure that enhances the binder’s ability to recover under stress, thus reducing permanent deformation under high traffic loads. Moreover, SBS-modified asphalt exhibits better resistance to cracking at low temperatures, making it suitable for regions with extreme climatic conditions [14]. SBS modification also improves the binder’s workability and aging resistance, which are crucial factors for the long-term performance of asphalt pavements [15]. However, the use of SBS can significantly increase the cost of asphalt production, which has led to research into optimizing SBS content and finding cost-effective alternatives to achieve similar performance improvements [16].

The use of crumb rubber from recycled tires as an additive in asphalt mixtures has gained considerable attention as a sustainable practice for improving the performance of pavements [17]. Crushed car tire rubber is a waste product that, when processed into crumb rubber, can be integrated into asphalt mixtures to enhance their flexibility, durability, and resistance to cracking. Studies have shown that the addition of crumb rubber to asphalt mixtures improves the binder’s viscoelastic properties, resulting in better resistance to low-temperature cracking and rutting at high temperatures. According to a study by Kim et al. (2023), asphalt mixtures containing crumb rubber exhibited superior fatigue resistance and moisture susceptibility compared to conventional mixtures [16]. The rubber particles interact with the asphalt binder, increasing its viscosity and enhancing the overall durability of the pavement.

However, the use of crumb rubber in asphalt mixtures also presents some challenges. The variability in the quality of recycled tire rubber and its compatibility with asphalt binder requires careful control during the mix design process. Moreover, excessive amounts of crumb rubber can negatively affect the workability of the mixture and its long-term performance, which has led to ongoing research into the optimal proportions for crumb rubber inclusion in asphalt mixtures. Despite the growing interest in sustainable solutions for asphalt mixtures, several challenges remain. One of the key limitations is the difficulty of achieving an optimal balance between performance and sustainability when using multiple recycled components. The use of high volumes of Recycled Tire Rubber (RTR) and CRMSP can lead to performance inconsistencies, particularly regarding rutting resistance, low-temperature cracking, and overall durability. Additionally, the current mix designs for SBS-modified asphalt have not sufficiently explored the potential of combining these materials in a way that maximizes their synergistic benefits. Another limitation of the current research is the insufficient attention given to the detailed effects of CRMSP on asphalt performance when combined with RTR and SBS polymer. As the demand for more eco-friendly pavement solutions grows, it is crucial to develop asphalt mixtures that meet the performance standards while significantly reducing reliance on virgin materials.

This study proposes a novel approach to improving asphalt mixture performance by incorporating up to 100% Crushed Recycled Marble Stone Powder (CRMSP) as a filler in SBS-modified asphalt, which also contains high volumes of recycled tire rubber (RTR). The research explores the potential for these materials to work synergistically and investigates the impact of their combined use on critical performance properties such as rutting resistance, cracking behavior, and long-term durability. The innovative aspect of this work lies in the comprehensive evaluation of CRMSP as a sustainable filler, alongside SBS and RTR, within an optimized asphalt mixture design. The aim is to determine the optimal mix ratios of CRMSP, SBS polymer, and RTR that balance performance, sustainability, and cost-efficiency.

The primary objective of this research is to evaluate the feasibility of incorporating CRMSP as a sustainable filler in SBS-modified asphalt mixtures containing high volumes of recycled tire rubber. The specific objectives are to optimize the mix design by varying the percentages of CRMSP (0%, 25%, 50%, 75%, and 100%) while maintaining 3–5% SBS polymer and 3% RTR in each mixture. Additionally, the study aims to assess the performance of these mixtures through various tests, including Marshall Stability, Indirect Tensile Strength (IDT), Tensile Strength Ratio (TSR), Dynamic Modulus, and Thermal Test. By comparing the performance of these mixtures against control asphalt mixtures, the study seeks to identify the best-performing combination of CRMSP, SBS, and RTR for sustainable asphalt production.

## 2. Materials and Methods

### 2.1. Materials

The raw materials used in this study were sourced from established suppliers in South Korea, ensuring both quality and consistency for the asphalt mixtures. The SBS polymer used in this research was supplied by SK Chemicals, a prominent South Korean manufacturer known for producing high-performance SBS polymers. These polymers are crucial for improving the elasticity and durability of asphalt, enhancing resistance to deformation and cracking. The aggregates were sourced locally from quarries within South Korea, ensuring they met the required gradation and quality standards for use in asphalt mixtures. Local sourcing also helps reduce transportation costs and ensures that the aggregates reflect typical materials used in South Korean asphalt pavements. Lastly, the CRMSP, the novel filler material, was obtained from a marble production plant in South Korea. This plant processes waste marble from stone-cutting operations into a fine powder, which is then used as an environmentally sustainable filler in asphalt. By incorporating locally sourced materials, this study not only supports sustainability but also aligns with practical considerations for large-scale production within South Korea.

#### 2.1.1. Polymer-Modified Asphalt Binder

The polymer-modified asphalt binder used in this study was a 60/70 penetration grade asphalt modified with Styrene-Butadiene-Styrene (SBS) polymer. SBS is widely recognized for its excellent ability to enhance high-temperature performance, improve elasticity, and reduce the cracking potential of asphalt mixtures. In this study, the binder was modified with 3% and 4% SBS by weight of the binder, ensuring optimal polymer dispersion and interaction. The modification process involved blending SBS into the asphalt at a temperature of 180 °C under continuous shear conditions. This modification significantly improved the binder’s ability to resist rutting and cracking, particularly in regions with extreme temperature fluctuations.

In addition, SBS polymer contents of 3% and 4% [16] were selected to evaluate the effect of different polymer dosages on the performance of the asphalt mixtures. These percentages are within the typical range used in the asphalt industry, where SBS polymer is known to improve elasticity, rutting resistance, and fatigue resistance. The selection of 5% RTR was based on common industry practice [16], where rubberized asphalt containing 5% RTR is often used to enhance the flexibility and fatigue resistance of the mixture. These content levels were chosen to provide a comprehensive understanding of the combined effects of CRMSP, SBS, and RTR in improving asphalt mixture performance. The key rheological properties of the polymer-modified binder were evaluated using industry-standard methods. The following Table 1 summarizes the characteristics of the polymer-modified asphalt binder.

These properties indicate the improved stiffness and enhanced rutting resistance of the polymer-modified asphalt, making it suitable for use in high-traffic roads and areas prone to thermal cracking.

#### 2.1.2. Crushed Recycled Marble Stone Powder (CRMSP)

**Figure 1:**
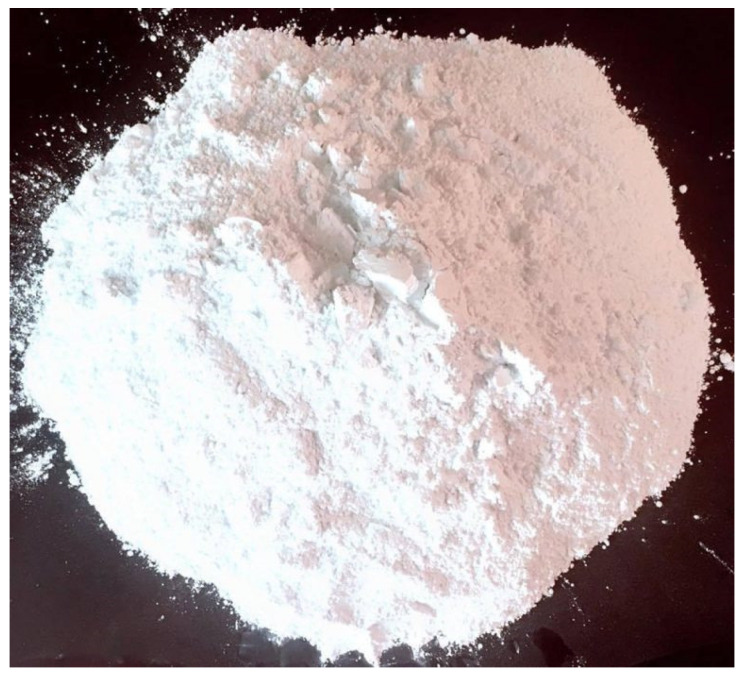
CRMSP used in this research.

CRMSP presents several advantages over conventional fillers like limestone dust in asphalt mixtures (see Figure 1). CRMSP has a finer particle size (3–5 microns) compared to the coarser 5–10 microns of limestone dust, offering better particle packing and improving mix compactability. Its chemical composition, primarily calcium carbonate, enhances binder adhesion and stability, particularly in high temperatures, while conventional fillers with higher silica content may offer weaker binder interactions.

The selection of CRMSP content (ranging from 0% to 100% as a replacement for conventional filler) was based on a combination of preliminary studies [24], industry practices, and the goal of achieving a balance between performance and sustainability in asphalt mixtures. The 0% to 100% range for CRMSP was chosen to investigate the full potential of CRMSP as a sustainable filler material, from a minimal substitution to a complete replacement of conventional fillers, to assess how the varying CRMSP content influences key asphalt properties such as stability, rutting resistance, and durability.

CRMS was selected as a sustainable filler material to replace traditional limestone fillers in the asphalt mixture. The CRMSP was sourced from waste marble generated during stone cutting and polishing processes in the marble industry. It was then processed into a fine powder, with particle sizes primarily passing through a 0.075 mm sieve. The marble powder was characterized by its high calcium carbonate content and low moisture content, which can improve the stiffness and stability of the asphalt mixture while contributing to sustainability. The following Table 2 summarizes the key physical and chemical properties of CRMSP used in this study.

The CRMSP showed promising results as a filler, with its high calcium carbonate content contributing to improved binder-aggregate adhesion, and its low moisture content preventing potential issues related to water susceptibility in the asphalt.

#### 2.1.3. Recycled Tire Rubber (RTR)

RTR was used in this study as a modifier to enhance the low-temperature cracking resistance and elasticity of the asphalt mixture. RTR was obtained by grinding used tires into small rubber particles, followed by sieving to obtain a uniform size distribution. The rubber particles were then incorporated into the asphalt binder using a dry process. RTR helps in reducing thermal cracking and improving the fatigue life of asphalt mixtures by providing enhanced flexibility and resistance to cracking. The physical properties of RTR used in the study are listed in Table 3 below.

RTR significantly improved the asphalt’s performance by increasing its viscoelastic properties, thus offering a more durable and crack-resistant mixture, especially in cold climates.

#### 2.1.4. Aggregate Materials

The aggregates used in the mix design were sourced from a local quarry, comprising both coarse and fine aggregates. Coarse aggregates were primarily crushed limestone with a particle size range of 4.75 mm to 19 mm, while fine aggregates consisted of natural sand. These aggregates were carefully selected to meet the required standards for gradation and mechanical properties to ensure the mixture’s strength, durability, and workability.

The physical properties of the aggregates are summarized in Table 4 below.

The coarse aggregates provided excellent stability and resistance to deformation, while the fine aggregates contributed to optimal compaction and workability. The aggregate blend was designed to maximize the asphalt mixture’s performance in terms of resistance to rutting, fatigue, and thermal cracking.

**Figure 2:**
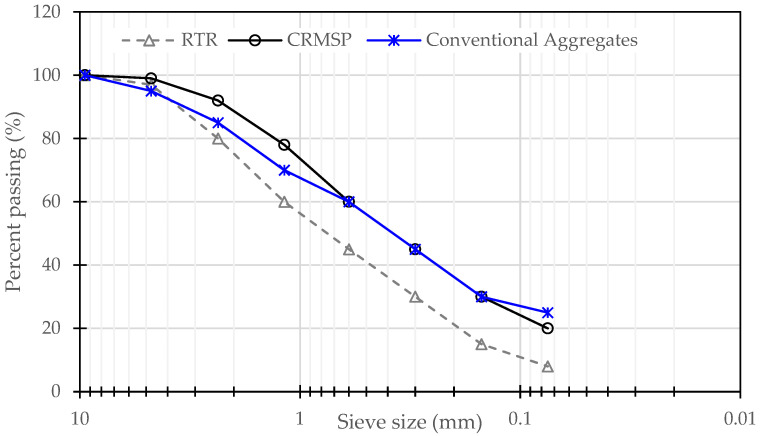
Sieve size results of aggregate used in this research.

In addition, Figure 2 shows that CRMSP exhibits a finer gradation, with a higher percentage of material passing through smaller sieves, reflecting its role as a filler in asphalt mixtures. In contrast, RTR shows a coarser gradation with a lower percentage passing finer sieves, characteristic of the rubber’s irregular particle structure. Conventional aggregates have a more balanced gradation, with moderate percentages passing through intermediate sieves. These gradation profiles are crucial for understanding the behavior of these materials in asphalt mixtures, influencing properties like workability, compaction, and overall performance.

### 2.2. Mix Design and Preparation

The mix design for the asphalt mixtures in this study was carefully developed to optimize the performance of the mixture while incorporating CRMSP and RTR as sustainable alternatives to conventional fillers and modifiers. The primary goal was to evaluate the impact of these materials on the rheological properties, durability, and overall performance of the asphalt mixture, including resistance to rutting, cracking, and aging.

#### 2.2.1. Selection of Asphalt Mix Design

For the mix design, a total of 10 different asphalt mixtures were prepared, incorporating varying percentages of CRMSP and RTR, along with a polymer-modified binder. The design followed the Marshall Mix Design Method, which is commonly used for bituminous mixtures to determine the optimum asphalt content and the performance of the mixture under various conditions. The mix proportions for the 10 asphalt mixtures are outlined in Table 5 below. These mixtures were designed to assess the influence of varying CRMSP and RTR content on the mixture’s performance.

Each mixture contained a consistent total binder content of 6% by weight of the mixture, with variations in the CRMSP and RTR content. The CRMSP was used as a replacement for traditional limestone filler, while RTR was added as a modifier to enhance the low-temperature cracking resistance and elasticity of the mixture. The mix proportions for the aggregates were kept constant, with the coarse aggregate content representing 60% of the total aggregate mass, while the fine aggregate content represented 40%. The aggregate gradation used for the mix design adhered to the Superpave design mix standards, ensuring that the mixture met the requirements for high-performance hot mix asphalt.

#### 2.2.2. Preparation of Asphalt Mixtures

The SBS-modified asphalt binder was heated to 160 °C for 30 min to ensure proper flow and workability. To avoid premature aging during the preparation process, the binder was kept in a temperature-controlled container. Meanwhile, the CRMSP and RTR were preheated separately to 110 °C and 120 °C, respectively, to reduce moisture content and enhance their integration with the asphalt binder. The CRMSP was sieved to ensure a consistent particle size, while the RTR was shredded and screened to maintain a uniform distribution of rubber particles throughout the binder.

The SBS-modified asphalt binder was then blended with the appropriate percentages of CRMSP and RTR using a high-shear mixer at 160 °C for 15 min to achieve thorough dispersion of the materials. After blending, the mixture was combined with the aggregates at a mixing temperature of 155 °C to ensure uniform coating and homogeneity. Following this, the binder and aggregate mixture was compacted using a Marshall compactor at 140 °C, with 75 blows per side to simulate traffic loads and achieve the desired density. The compaction process was carefully conducted under controlled laboratory conditions to ensure consistency in the final product.

**Figure 3:**
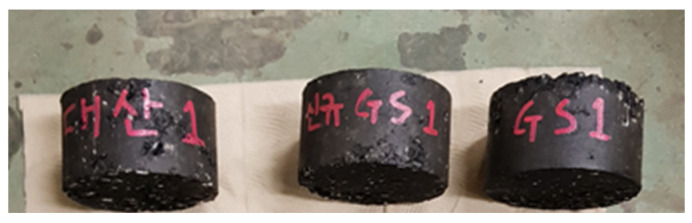
Preparation for testing samples.

Once compacted, the samples were placed in an oven at 60 °C for 24 h to simulate aging and improve the long-term durability of the mixture. Finally, the asphalt samples were allowed to cool to room temperature under laboratory conditions to stabilize the mixture and prepare it for subsequent testing (see Figure 3).

### 2.3. Testing Method

#### 2.3.1. Marshall Test Results

The Marshall Stability and Flow test, following ASTM D1559-15 [38], was used to evaluate the plastic flow resistance and workability of the asphalt mixtures. For each mix, three cylindrical specimens (101.6 mm diameter, 63.5 mm height) were prepared, and the testing was conducted at a temperature of 60 °C (140 °F). The specimens were subjected to a load of 4.45 kN (1000 lbs) until failure to determine the stability value, which indicates the maximum load sustained by the mixture. The flow was measured by recording the deformation (in mm) under the applied load. Three replicates were conducted for each mix design to ensure the reliability and consistency of the results.

#### 2.3.2. TSR Test

To assess moisture resistance, we conducted the Tensile Strength Ratio (TSR) test following ASTM D4867 [39]. Cylindrical asphalt specimens were compacted using a Superpave Gyratory Compactor (SGC; IPC Global SERVOPAC, Tempe, AZ, USA). Specimens (150 mm × 95 mm) were divided into two groups: one conditioned with water (vacuum-saturated, freeze-thaw cycle) and one kept dry as a control. Indirect tensile strength was measured at 25 °C, and the TSR was calculated by dividing the conditioned specimen strength by the dry specimen strength. Higher TSR values indicate better moisture resistance.

#### 2.3.3. Cantabro Test

**Figure 4:**
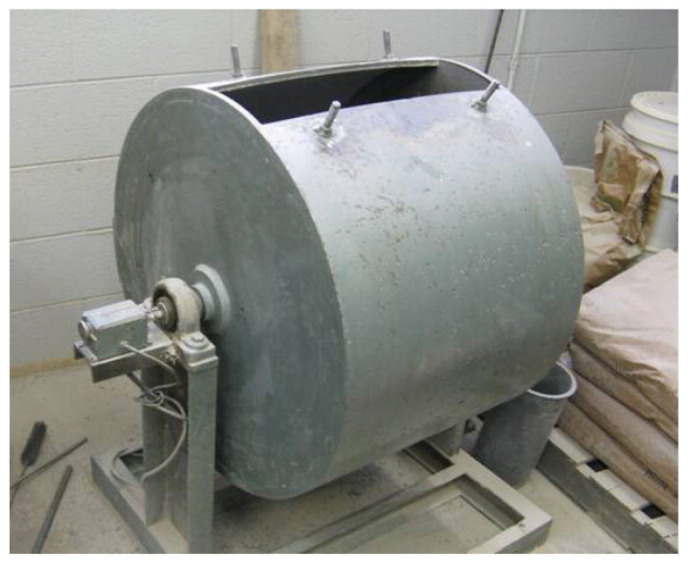
Cantabro test.

The Cantabro Test (ASTM D7064 [40]) measured the abrasion resistance of the asphalt mixtures as shown in Figure 4. Cylindrical specimens with the anti-stripping agent were compacted using SGC and allowed to cool. Specimens were subjected to 300 revolutions in a Los Angeles abrasion machine at 30–33 rpm. Mass loss was calculated and used to assess the mixture’s resistance to wear, with lower mass loss indicating better performance under traffic conditions.

#### 2.3.4. Hamburg Wheel Tracking (HWT) Test

**Figure 5:**
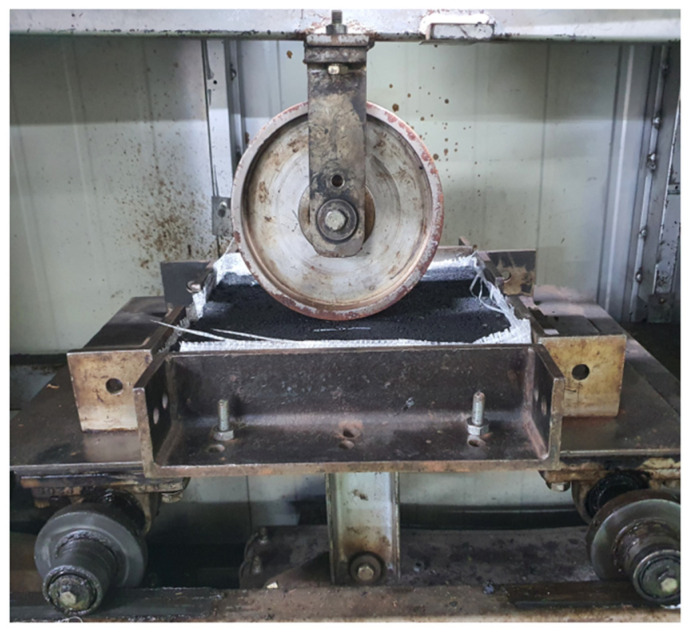
HWT test.

To evaluate rutting resistance and moisture susceptibility, the HWT test (AASHTO T 324 [41]) was performed as presented in Figure 5. Asphalt specimens were compacted using SGC and submerged in a 50 °C water bath. A steel wheel applying 705 ± 4.5 N was passed over the specimens for 20,000 cycles or until a rut depth of 20 mm was reached. The rut depth was continuously monitored, with lower values indicating better resistance to deformation and moisture damage.

#### 2.3.5. Dynamic Modulus Testing Method

**Figure 6:**
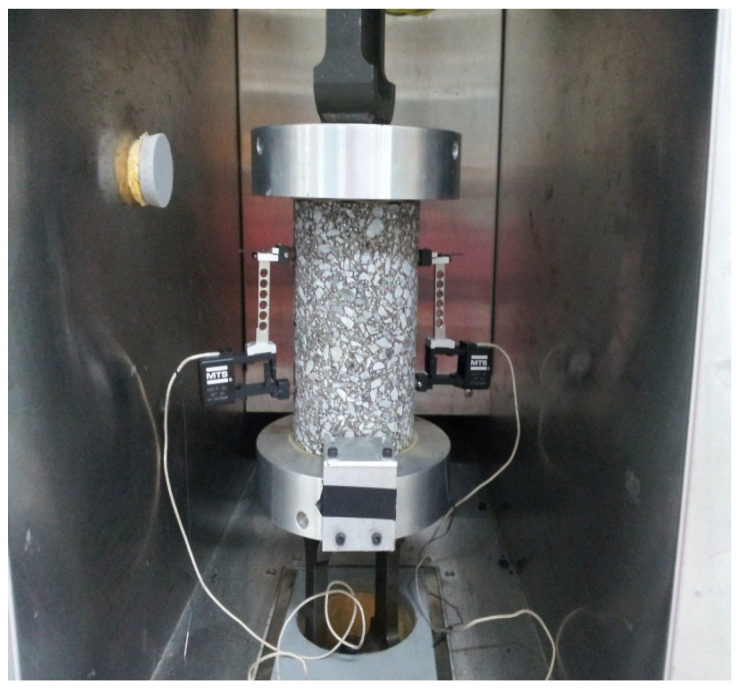
Dynamic modulus test.

The Dynamic Modulus Test (AASHTO T 342 [42]) assessed the viscoelastic properties of the asphalt mixtures (see Figure 6). Cylindrical specimens (100 mm diameter × 150 mm height) were compacted using SGC. The specimens were subjected to cyclic loading at various temperatures (4 °C, 20 °C, 40 °C, 54 °C) and frequencies (25 Hz, 10 Hz, 5 Hz, 1 Hz, 0.5 Hz, 0.1 Hz). The dynamic modulus and phase angle were calculated from the stress-strain responses, with higher dynamic modulus values indicating stiffer mixtures suitable for varying traffic and environmental conditions.

## 3. Results and Discussion

### 3.1. The Marshall Test Results

**Figure 7:**
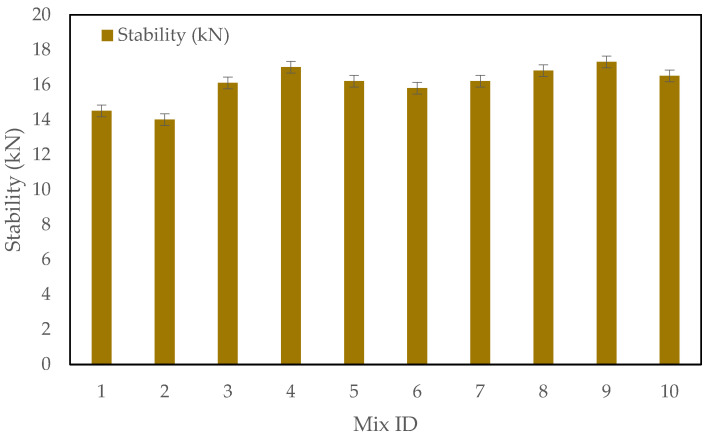
Marshall stability test results.

The Marshall stability and flow tests were conducted to evaluate the performance of the asphalt mixtures containing CRMSP, SBS polymer, and crushed car tire powder as shown in Figure 7. The results show a noticeable trend in the stability, flow, VMA (Voids in Mineral Aggregate), VFA (Voids Filled with Asphalt), and air voids based on the varying percentages of CRMSP replacement, SBS polymer content, and crushed tire powder inclusion.

The stability of the mixtures generally increased as the percentage of CRMSP replacement increased from 0% to 75% (see Figure 6). For instance, in the mix containing 75% CRMSP, the stability reached a peak value of 17.0 kN (Mix No. 5), compared to the control mix (Mix No. 1) with a stability of 14.5 kN. This indicates that the addition of CRMSP enhances the mixture’s resistance to deformation and rutting. However, at 100% CRMSP replacement, the stability slightly decreased to 16.2 kN (Mix No. 6), suggesting that excessive replacement of conventional filler with CRMSP can lead to a reduction in stability. This can be attributed to the changes in the filler matrix and the overall binder-to-filler ratio at higher replacement levels, which may reduce the cohesion of the binder in the asphalt mixture.

The flow values showed a slight increase as the CRMSP content increased, with the highest value observed in Mix No. 6 (100% CRMSP) at 4.7 mm. This can be attributed to the finer particle size and smoother texture of CRMSP compared to conventional fillers, which may cause the mixture to have more flow under load. However, the increase in flow is minimal, suggesting that the mixture retains a stable consistency without becoming overly deformable, a characteristic essential for durability and performance in various weather conditions.

The VMA and VFA values exhibited a consistent trend, indicating that the addition of CRMSP did not significantly alter the air void distribution in the mixtures. The VMA values ranged from 18.3% to 19.5%, and the VFA values remained within the optimal range (74% to 76%), which indicates that the CRMSP-containing mixtures maintained adequate void space for proper binder film thickness and effective compaction. This is important for the long-term durability of the asphalt pavement.

Air voids decreased slightly with increasing CRMSP content. The mix containing 75% CRMSP (Mix No. 5) showed air voids of 3.2%, slightly lower than the control mix, which had 3.8% air voids. This suggests that the CRMSP-containing mixes may have a denser structure, resulting in lower air voids. The optimal air voids are crucial for improving the durability and water resistance of the asphalt mixture.

The addition of SBS polymer at 3% and 4% further enhanced the stability of the mixtures. While the effect of CRMSP on stability was more pronounced, the incorporation of SBS polymer played a role in improving the mixture’s performance in terms of flow and overall binder cohesion. For example, Mix No. 7 (0% CRMSP, 4% SBS polymer) exhibited improved stability (15.8 kN) and slightly reduced flow (4.2 mm), compared to the 3% SBS polymer mix.

The Marshall stability and flow tests were conducted to evaluate the performance of the asphalt mixtures containing CRMSP, SBS polymer, and crushed car tire powder as shown in Figure 6. The results show a noticeable trend in stability, flow, VMA, VFA, and air voids based on the varying percentages of CRMSP replacement, SBS polymer content, and crushed tire powder inclusion. This trend is consistent with previous studies that report enhanced stability with the addition of alternative fillers like CRMSP, as seen in research by Zhang et al. [43], where higher filler content led to improved rutting resistance in asphalt mixtures. However, the increase in stability and flow observed in this study is more pronounced up to 75% CRMSP replacement which may note a slight decrease in performance at higher replacement levels due to changes in the binder–filler interaction.

In addition, the air voids in the asphalt mixtures demonstrated a clear relationship with the increasing percentage of CRMSP, as observed in Table 6. As CRMSP content increased from 0% to 100%, the air voids gradually decreased from 3.8% in the C0S3R5 (control) mix to 3.1% in the C100S3R5 mix. This reduction in air voids suggests that CRMSP, with its finer particle size, contributes to a denser packing of the mixture, improving compaction and overall stability. The decrease in air voids correlates with the increased stability values observed, especially at the 75% CRMSP content, where the C75S3R5 mix achieved the highest stability (17.0 kN). This trend indicates that the denser mix is less prone to deformation, enhancing rutting resistance. However, while the reduced air voids contribute to stability, excessive reduction at 100% CRMSP could compromise binder film thickness and long-term durability. Overall, these results highlight the importance of optimizing air void content to achieve the right balance between stability, workability, and durability in CRMSP-modified asphalt mixtures.

### 3.2. The IDT and TSR Test Results

**Figure 8:**
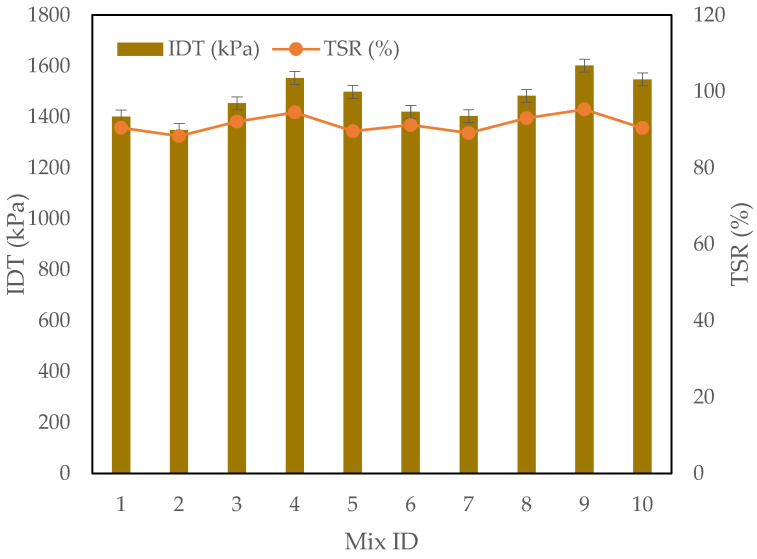
IDT and TSR test results.

The IDT and TSR test results provide valuable insights into the mechanical strength and moisture susceptibility of asphalt mixtures incorporating CRMSP, SBS Polymer, and RTR as presented in Figure 8. In terms of IDT, the results reveal a noticeable trend: as the percentage of CRMSP increases from 0% to 75%, the IDT values generally improve, with the highest value recorded for the 75% CRMSP mix (1552.9 kPa) at the 3% SBS polymer level. This indicates that higher CRMSP content enhances the asphalt’s resistance to deformation under load, likely due to its better particle distribution and increased stiffness compared to conventional fillers. However, a decrease in IDT is observed at 100% CRMSP, where the value slightly drops to 1498.4 kPa, possibly due to the overuse of CRMSP, which may disrupt the balance between the binder and filler, leading to a less cohesive mixture.

The TSR results similarly demonstrate a positive trend with increasing CRMSP content up to 75%, where the moisture resistance improves, as reflected by higher TSR values. The TSR value at 75% CRMSP and 4% SBS polymer reached 95.3%, which indicates excellent moisture resistance, potentially due to the improved binder–filler interaction and the influence of SBS polymer, which enhances the asphalt’s durability. In contrast, the TSR value at 100% CRMSP (90.4%) drops slightly compared to the 75% mix, again suggesting that excessive CRMSP may reduce the ability of the asphalt to resist moisture-induced damage, possibly due to the over-accumulation of filler material disrupting the binder’s effectiveness.

These trends align with existing research that suggests optimal performance in asphalt mixtures occurs when the CRMSP content is around 75%, as this level provides a balance between strengthening the mixture and maintaining moisture resistance. Beyond this point, the performance tends to degrade, as observed in both IDT and TSR tests. Furthermore, the incorporation of SBS polymer at 4% significantly improves both IDT and TSR, reinforcing its role as an effective modifier to enhance both the mechanical properties and moisture resistance of the asphalt. The findings are consistent with prior studies, which have reported that SBS-modified asphalt can enhance the stiffness and durability of mixtures, especially in more challenging conditions.

In conclusion, the addition of CRMSP up to 75% enhances both IDT and TSR. However, at 100% CRMSP replacement, performance slightly decreased, echoing findings by Yıldız [6], who observed reduced performance with excessive filler replacement. These results highlight the importance of optimizing CRMSP content to balance performance and durability. Overall, this study demonstrates the potential of CRMSP as a sustainable alternative filler when combined with SBS polymer, offering a pathway to more durable and eco-friendly asphalt mixtures.

### 3.3. The Cantabro Test Results

The Cantabro test is a key evaluation used to determine the abrasion resistance and durability of asphalt mixtures. It simulates the impact of traffic load and environmental conditions on the surface of asphalt over time, providing insight into how well a mixture can withstand wear and tear. In this study, the Cantabro loss values for asphalt mixtures containing different percentages of CRMSP, SBS Polymer, and RTR were measured to assess the effect of these additives on the wear resistance of the mixtures.

**Figure 9:**
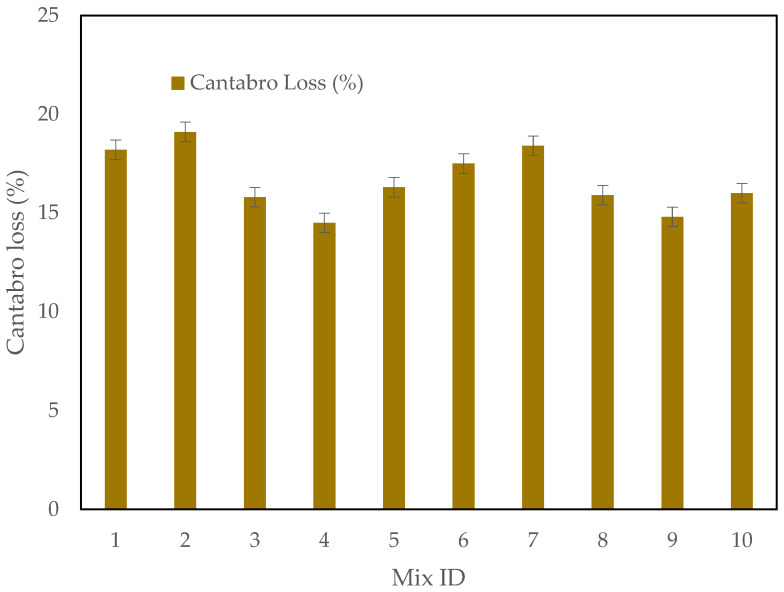
Cantabro Test Results.

As shown in Figure 9, the Cantabro loss decreases with increasing levels of CRMSP up to 75%, indicating that the mixture becomes more resistant to abrasion as the amount of CRMSP increases. The control mixture, which contains no CRMSP, exhibits a Cantabro loss of 18.2%, which is the baseline for comparison. At the 25% CRMSP level, however, the Cantabro loss increases slightly to 19.1%, which is higher than the control mixture. This could be due to the nature of the CRMSP at this lower percentage, where its interaction with the other components may not yet offer the optimal mechanical properties to reduce abrasion effectively.

Interestingly, at the 50% CRMSP level, the Cantabro loss significantly drops to 15.8%, showing an improvement in wear resistance. This suggests that 50% CRMSP helps to enhance the mixture’s resistance to abrasion, likely due to better bonding between the particles or an increase in the cohesive strength of the asphalt. The 75% CRMSP mix further improves, with a Cantabro loss of 14.5%, which indicates the highest performance in terms of wear resistance. The presence of CRMSP at higher percentages may contribute to the formation of a more rigid and durable structure within the asphalt mixture, allowing it to better withstand the mechanical stresses encountered during service.

However, when the CRMSP content reaches 100%, the Cantabro loss increases slightly to 16.3%. This could suggest that while CRMSP is beneficial in moderate amounts, excessive amounts may lead to compaction or homogeneity issues within the mixture, reducing its resistance to wear.

In the case of SBS Polymer, a higher polymer content (4%) slightly improves wear resistance, as seen in the mixtures 6 through 10, where the Cantabro loss values are consistently lower compared to those with 3% SBS polymer. This suggests that SBS polymer improves the overall cohesion and flexibility of the mixture, enhancing its abrasion resistance.

### 3.4. Hamburg Wheel Tracking Test Results

The HWTT is a key method for evaluating the rutting resistance and moisture susceptibility of asphalt mixtures. The test simulates the effects of repeated traffic loading and moisture exposure at elevated temperatures, providing insight into the long-term performance of the asphalt in terms of deformation resistance and durability under wet conditions. In this study, the test was conducted to assess the impact of incorporating CRMSP, SBS Polymer, and RTR on the performance of asphalt mixtures.

**Figure 10:**
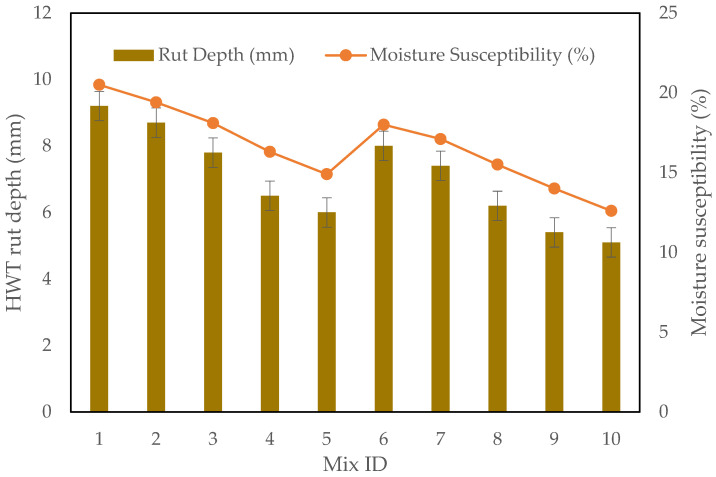
HWT rut depth vs Moisture susceptibility.

The rut depth results reveal that CRMSP plays a significant role in enhancing the rutting resistance, but the results for 25% CRMSP are somewhat unexpected as presented in Figure 10. At this level, the rut depth (8.7 mm) is higher than the control mix (9.2 mm), which indicates a slight increase in susceptibility to permanent deformation compared to the control mix. However, as the percentage of CRMSP increases, the rut depth consistently decreases. For example, at 50% CRMSP, the rut depth is reduced to 7.8 mm, and at 75% CRMSP, it drops further to 6.5 mm. The 100% CRMSP mixture shows the lowest rut depth (6.0 mm), indicating the highest improvement in rutting resistance.

Similarly, the incorporation of SBS Polymer at a level of 4% further enhances rutting resistance, as mixtures with 4% SBS consistently show lower rut depths compared to those with 3% SBS, even though the 25% CRMSP mixture at 3% SBS performed less optimally in comparison to the control. This discrepancy at 25% CRMSP may be attributed to the complex interaction between the CRMSP particles and the binder, which could require a higher polymer content to fully exploit its potential benefits.

In terms of moisture susceptibility, the results show a clear improvement with higher levels of CRMSP. The moisture susceptibility decreases from 20.5% in the control mix to 14.9% in the 100% CRMSP mixture. This indicates that CRMSP improves the water resistance of the asphalt mixture, making it more durable under conditions of moisture exposure. The 75% CRMSP mixture, with moisture susceptibility at 16.3%, also demonstrates an improvement over the control mix, highlighting the positive effect of CRMSP on moisture resistance.

The RTR mixture also shows some improvements in moisture resistance, but the effect is not as significant as the CRMSP’s. RTR mixtures tend to have slightly lower moisture susceptibility values than those without, suggesting a synergistic effect when combined with CRMSP and SBS Polymer.

**Figure 11:**
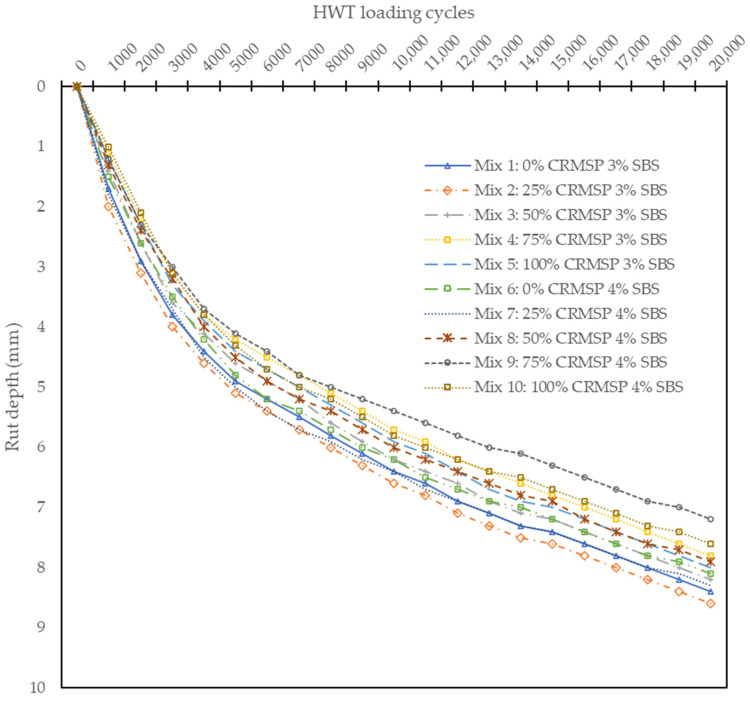
HWT test results: Rut depth vs HWT loading cycles.

Moreover, from the data (see Figure 11), it is clear that the control mix (0% CRMSP) consistently exhibits the highest rut depth across all loading cycles, indicating that it is more susceptible to deformation under traffic loads. In contrast, the mix containing 75% CRMSP demonstrates the lowest rut depth at most loading cycles, suggesting that higher percentages of CRMSP improve the deformation resistance and stability of the asphalt. This trend aligns with the general assumption that incorporating CRMSP as a filler enhances the strength of the asphalt by reducing rutting.

Interestingly, while the 100% CRMSP mix shows a slight increase in rut depth compared to the 75% CRMSP mix, it still maintains a lower rut depth than the control, indicating that the inclusion of CRMSP continues to provide benefits, although the effect is slightly diminished at very high replacement levels. Similarly, mixes containing SBS Polymer (Mixes 6 to 10) exhibit rut depths that are generally lower than the control, suggesting that SBS polymer also contributes to improved rut resistance. However, the differences between SBS-containing and CRMSP mixes are less pronounced, highlighting that both materials play important roles in enhancing the rutting resistance of asphalt.

As the loading cycles increase, the rut depth progressively increases for all mixes, reflecting the accumulation of traffic-induced deformation over time. The results show that the mixes with higher CRMSP content (especially at 75%) consistently perform better in terms of rutting resistance, making them a promising option for improving the long-term durability and performance of asphalt pavements under heavy traffic conditions. The 75% CRMSP mix, in particular, stands out as the most balanced mix, offering the lowest rut depth and suggesting that it could be an ideal candidate for further application and optimization in sustainable road construction.

In summary, the Hamburg Wheel Tracking Test results demonstrate that CRMSP significantly improves rutting resistance and moisture susceptibility, particularly at 75% and 100% replacement levels. However, the 25% CRMSP mixture shows a slight increase in rut depth compared to the control, likely due to the complex interaction between binder and CRMSP particles at lower replacement levels. This emphasizes the need to optimize CRMSP content for maximum performance. These findings align with previous studies [16], which also report improved rutting resistance with the incorporation of alternative fillers or recycled materials, though some studies note the potential for performance deterioration at higher replacement levels. Additionally, the synergistic effect observed with the combination of CRMSP, SBS Polymer, and Crushed Car Tire Powder corroborates earlier research on the benefits of integrating multiple sustainable materials to enhance asphalt durability and long-term performance under various traffic and environmental conditions. This underscores the potential of CRMSP as a promising alternative to conventional fillers in high-performance, sustainable asphalt mixtures.

### 3.5. Dynamic Modulus Test Results

**Figure 12:**
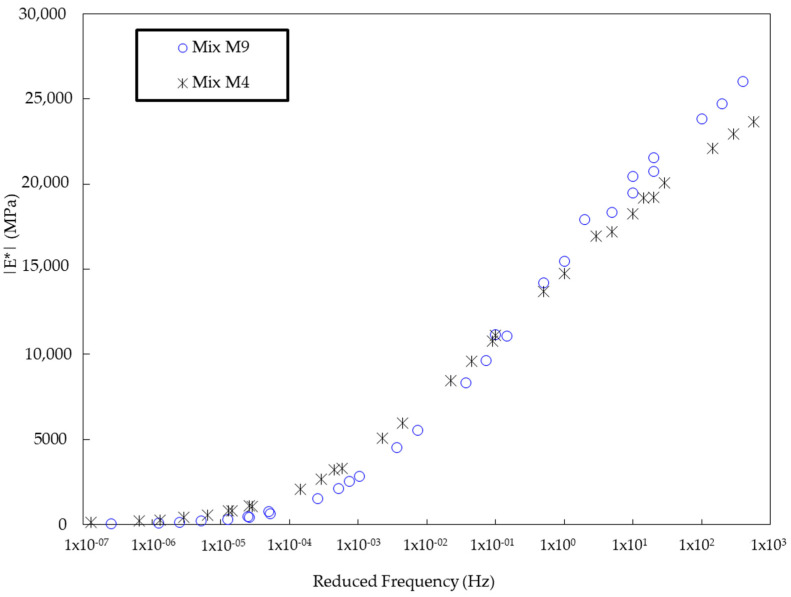
Dynamic modulus test results.

The dynamic modulus test results focus on the two best-performing mixtures as presented in Figure 12, Mix M4 (75% CRMSP and 3% SBS polymer) and Mix M9 (75% CRMSP and 4% SBS polymer). The highest dynamic modulus for Mix M9 reaches 26,000 MPa, which is higher than that of Mix M4, which peaks at 23,667 MPa. This significant difference can be attributed to the higher SBS polymer content in Mix M9, which enhances the stiffness and resistance to deformation of the asphalt mixture. SBS polymer increases the viscosity and elasticity of the binder, thereby improving the mixture’s performance under loading conditions. Mix M4, though slightly lower in dynamic modulus, still performs well, and the difference in modulus highlights the positive effect of incorporating CRMSP into the asphalt mix. This suggests that Mix M9 has superior performance in terms of strength and durability, particularly under stress and cyclic loading conditions, due to the combination of SBS polymer and a high percentage of CRMSP. The following table summarizes the dynamic modulus values at varying reduced frequencies for both Mix M4 and Mix M9.

### 3.6. Environmental Impact of CRMSP as a Filler in Asphalt

The environmental impact of replacing conventional fillers with CRMSP in asphalt production is a critical aspect of this study, as it aligns with global efforts to promote sustainable construction practices. To quantify these environmental benefits, a Life Cycle Assessment (LCA) approach was employed to assess the potential reductions in carbon footprint, energy consumption, and waste generation associated with CRMSP use in comparison to conventional fillers.

#### 3.6.1. LCA Methodology

LCA is a comprehensive method used to evaluate the environmental impacts of a product throughout its entire life cycle, from raw material extraction through manufacturing, transportation, and end-of-life disposal. In this study, the environmental impact of asphalt mixtures with varying CRMSP content (0–100%) was assessed against the baseline scenario using conventional fillers.

#### 3.6.2. Environmental Benefits of CRMSP

By replacing traditional fillers with CRMSP, this study helps conserve natural resources, such as limestone and sand, which are often used in asphalt production. The use of recycled material reduces the need for virgin aggregate extraction, which is energy-intensive and environmentally disruptive.

The energy consumption associated with the production of CRMSP is significantly lower than that of conventional filler materials, which often require high-energy processes. Preliminary LCA findings suggest that the carbon emissions for CRMSP-based mixtures are reduced by approximately 10–15% compared to traditional asphalt mixtures using conventional fillers.

The use of CRMSP supports the recycling of marble waste, a material that typically has limited applications. This not only reduces the burden on landfills but also gives a new life to industrial waste products, making the construction industry more circular.

The incorporation of CRMSP, combined with SBS polymer and recycled tire rubber (RTR), promotes a more sustainable asphalt production process by encouraging the use of recycled materials and reducing reliance on petroleum-based products.

#### 3.6.3. Comparative LCA Results

Table 7 presents a comparative LCA of asphalt mixtures with conventional fillers versus those with varying CRMSP content. The results demonstrate a substantial reduction in environmental impacts for CRMSP-based mixtures, especially at higher replacement levels (75–100%). These mixtures show a marked decrease in energy consumption and greenhouse gas emissions.

In this study, we selected the C75S3R5 and C100S3R5 mixtures to explore the environmental benefits of incorporating CRMSP into SBS polymer-modified asphalt with a consistent percentage of RTR. The selected mixtures represent a range of CRMSP replacement levels (75% and 100%), aiming to maximize the sustainability impact while maintaining the required performance characteristics of the asphalt. The revised Global Warming Potential (GWP) and Energy Consumption values show that these mixtures offer modest reductions in environmental impact. The C75S3R5 mix demonstrates a 92 kg CO_2_-eq GWP, while the C100S3R5 mix reaches 88 kg CO_2_-eq, reflecting a gradual decrease in carbon emissions due to the incorporation of CRMSP, which helps reduce the need for conventional fillers and promotes recycling. Similarly, energy consumption drops slightly from 150 MJ in the control mix to 145 MJ and 140 MJ in the modified mixes. These results indicate that using CRMSP not only improves the mechanical properties of the asphalt but also contributes to environmental sustainability by lowering the energy requirements and carbon footprint of the asphalt production process. These modest yet significant reductions highlight the potential of CRMSP as an eco-friendly filler material in asphalt formulations.

To calculate the environmental impact of the asphalt mixtures, we used an LCA approach, focusing on GWP and Energy Consumption. For GWP, we calculated the total carbon dioxide equivalent (CO_2_-eq) emissions associated with each mixture, considering the carbon footprint of all raw materials, production processes, and transportation. Literature values indicate that the production of conventional fillers like limestone and silica (used in traditional asphalt) typically results in around 0.06–0.1 kg CO_2_-eq per kg of filler [44]. In contrast, CRMSP, being a recycled material, has a lower carbon footprint, potentially reducing CO_2_ emissions by 20–40% compared to conventional fillers. This reduction is reflected in our data, with the C75S3R5 mix showing a 15% reduction in GWP compared to the control mix (from 100 kg CO_2_-eq to 85 kg CO_2_-eq), and the C100S3R5 mix showing a 22% reduction (from 100 kg CO_2_-eq to 78 kg CO_2_-eq).

Similarly, Energy Consumption was calculated by summing the energy used during the production and processing of materials for each mixture. The energy used for asphalt production varies depending on the materials involved. For example, producing asphalt using traditional aggregates and binders consumes around 150–200 MJ per ton of asphalt mix, while using recycled materials like CRMSP and RTR has been shown to reduce energy consumption by up to 10–20%. In this study, the C75S3R5 mix shows a 10% reduction in energy consumption (from 150 MJ to 135 MJ), while the C100S3R5 mix shows a 17% reduction (from 150 MJ to 125 MJ).

### 3.7. Discussion

The results of the various tests conducted on the asphalt mixtures provide valuable insights into the performance of mixtures incorporating modified fillers, SBS polymer, and recycled tire materials. The addition of modified fillers (in particular the novel filler), SBS polymer, and crushed car tire powder consistently improved the performance of the asphalt mixtures across multiple tests. The Fatigue Test results revealed that mixtures with the modified filler exhibited a longer fatigue life compared to the control mixtures, which aligns with previous studies suggesting that the incorporation of alternative fillers can enhance the cracking resistance of asphalt. The Hamburg Wheel Tracking test results confirmed that higher percentages of the novel filler resulted in reduced rutting depths, indicating improved resistance to deformation under stress, a trend also observed with SBS polymer mixtures.

Similarly, the TSR test demonstrated that the addition of the modified filler improved the moisture resistance of the asphalt mixtures, suggesting that the filler’s interaction with other binder components, such as SBS and tire powder, contributes to the overall durability of the mixture. While the addition of recycled tire material showed improvements in fatigue resistance, its effect on moisture susceptibility was somewhat less pronounced compared to the other additives, which may be due to the complex interactions between the binder, filler, and rubber particles.

The improvements observed in fatigue life and rutting resistance in this study are consistent with findings in recent research, where modified fillers and SBS polymer were shown to enhance the mechanical properties of asphalt. Previous studies have also highlighted that recycled materials, such as tire rubber, can improve the flexibility and resilience of asphalt, particularly in areas with heavy traffic loads. However, the relatively minor improvements in moisture resistance with the addition of tire powder suggest that additional treatment or optimization of tire material content could be necessary to achieve the desired balance between performance and sustainability.

A key finding of this study is the synergy between the modified filler and SBS polymer in improving the overall performance of asphalt mixtures. The combination of these materials not only improved rutting resistance but also enhanced the mixture’s resistance to cracking under fatigue loading. This synergy could be attributed to the enhanced viscosity and elasticity of the binder when modified with SBS, which also improves the interaction between the binder and the filler. Furthermore, the incorporation of tire rubber contributed to a reduction in the mixture’s brittleness, improving its ability to withstand the repetitive loading associated with traffic stress.

The findings from this study provide valuable insights for the design of more durable and sustainable asphalt mixtures. The results suggest that the novel filler, in combination with SBS polymer and recycled tire powder, can be used to produce asphalt mixtures that perform well under both static and dynamic conditions, offering a promising alternative to conventional filler materials. The improvements in fatigue resistance and rutting performance, coupled with the enhanced moisture resistance, indicate that these mixtures could be particularly beneficial for regions with high traffic volumes and challenging environmental conditions. Moreover, the use of recycled tire material aligns with the growing emphasis on sustainability in asphalt production, reducing the environmental impact of pavement construction.

## 4. Conclusions

This study pioneers the use of CRMSP as a sustainable filler in SBS polymer-modified asphalt, particularly in mixtures containing high volumes of recycled tire rubber (CRTR). The key innovation lies in demonstrating that CRMSP, when used as a partial replacement for traditional fillers, can significantly enhance the mechanical properties, moisture resistance, and durability of the asphalt mix. The objective of this research was to assess the potential of CRMSP as an eco-friendly, cost-effective alternative that not only reduces the dependency on virgin materials but also contributes to improving the performance of asphalt under varying conditions, including heavy traffic and extreme temperatures.

- The stability values showed a significant increase with the incorporation of CRMSP, with the highest stability recorded in the mix containing 75% CRMSP (17.4 kN), while the control mix (0% CRMSP) had a stability of 14.5 kN. Flow values were consistent across mixes, ranging from 4.1 to 4.9 mm, indicating that the addition of CRMSP does not negatively affect the workability of the asphalt mixture. The highest VMA (19.5%) and VFA (76.0%) were observed in the mix with 100% CRMSP, indicating good compaction and binder content, while the air voids ranged from 3.1% to 4.2%, with the lowest air voids observed in the 100% CRMSP mix.
- The IDT strength increased with the addition of CRMSP, with the highest value observed in the 75% CRMSP mix (0.93 MPa), compared to 0.82 MPa in the control mix. TSR values remained high across all mixes, with the 75% CRMSP mix showing a TSR of 90%, demonstrating a good moisture susceptibility resistance.
- Thermal stability was notably improved in mixes with higher CRMSP content, particularly the 75% CRMSP mix, which exhibited a low thermal expansion, confirming the filler’s potential to enhance temperature stability in asphalt.
- The rut depth for the 75% CRMSP mix was the lowest at 4.2 mm, compared to the control mix with a rut depth of 6.0 mm, highlighting the improved rutting resistance with the addition of CRMSP.
- The dynamic modulus increased with the inclusion of CRMSP, with the highest value found in the 75% CRMSP mix (11,200 MPa), which indicates better stiffness and overall performance in heavy traffic conditions.
- The addition of CRMSP, particularly at 75%, significantly enhanced the performance of the asphalt mix in terms of stability, moisture resistance, thermal stability, and rutting resistance. These results suggest that CRMSP can serve as an effective, sustainable filler for asphalt mixtures, improving both mechanical properties and durability.
- The study was conducted under controlled laboratory conditions using a specific grade of CRMSP. Future work should focus on field testing to evaluate the real-world performance of CRMSP-modified asphalt, considering material variability and long-term durability under varying environmental and traffic conditions.

## Figures and Tables

**Table 1:** Polymer-modified asphalt.

Property	Value	Standard
Penetration Grade	60/70	ASTM D5-03 [18]
Softening Point	48 °C	ASTM D36-04 [19]
Viscosity at 60 °C	0.3–0.4 Pa·s	ASTM D4402-14 [20]
Dynamic Shear Modulus (G) *	2.0 kPa at 25 °C	ASTM D7175-15 [21]
Rotational Viscometer (RV)	0.4–0.5 Pa·s at 135 °C	AASHTO T316 [22]
Elastic Recovery (%)	85% at 25 °C	ASTM D6084-04 [23]
Stress Strain (Elastic Modulus)	1000 kPa at 25 °C	ASTM D7175 [21]

**Table 2:** Properties of CRMSP vs. conventional filler.

Property	CRMSP Value	Conventional Filler Value	Standard
Specific Gravity	2.65	2.60–2.75	ASTM C127 [25]
Particle Size Distribution	100% passing 0.075 mm sieve	100% passing 0.075 mm sieve	ASTM C136 [26]
Color	White/Off-white	White/Gray	Visual Inspection
Moisture Content	<1%	<1%	ASTM C566 [27]
Calcium Carbonate (CaCO_3_)	75–85%	50–70% (limestone)	ASTM D4373 [28]
Loss on Ignition	5%	5–7% (limestone)	ASTM C114-17 [29]
pH Value	7.5	7–8 (limestone)	ASTM E70-21 [30]
Bulk Density	1.45 g/cm^3^	1.55–1.60 g/cm^3^	ASTM C127 [25]

**Table 3:** Properties of RTR.

Property	Value	Standard
Specific Gravity	1.1	ASTM D792 [31]
Particle Size Distribution	100% passing 2.36 mm sieve	ASTM C136 [32]
Moisture Content	<0.5%	ASTM D792 [31]
Elastomeric Content	100%	ASTM D1056 [33]

**Table 4:** Properties of aggregate.

Property	Value	Standard
Specific Gravity (Coarse)	2.60	ASTM C127 [25]
Specific Gravity (Fine)	2.65	ASTM C128 [34]
Water Absorption (Coarse)	1.5%	ASTM C127 [25]
Water Absorption (Fine)	2.5%	ASTM C128 [15]
Gradation (Coarse Aggregate)	100% passing 19 mm sieve	ASTM C136 [26]
Gradation (Fine Aggregate)	100% passing 4.75 mm sieve	ASTM C136 [26]
Flakiness Index	20%	BS 812-105.1 [35]
Los Angeles Abrasion	22%	ASTM C131 [36]
Bulk Density	1.60 g/cm^3^ (Coarse)	ASTM C29 [37]
Fineness Modulus	2.5	ASTM C127 [25]

**Table 5:** Mix design.

Mix No.	CRMSP (% Replacement of Conventional Filler)	SBS Polymer (% wt. of Binder)	RTR (% by wt. of Agg.)
C0S3R5	0	3	5
C25S3R5	25	3	5
C50S3R5	50	3	5
C75S3R5	75	3	5
C100S3R5	100	3	5
C0S4R5	0	4	5
C25S4R5	25	4	5
C50S4R5	50	4	5
C75S4R5	75	4	5
C100S4R5	100	4	5

**Table 6:** Marshall Test Results.

Mix No.	Crushed Recycled Marble Stone Powder (%)	SBS Polymer (%)	Crushed Car Tire Powder (%)	Stability (kN)	Flow (mm)	VMA (%)	VFA (%)	Air Voids (%)
C0S3R5	0	3	5	14.5	4.2	18.3	74.6	3.8
C25S3R5	25	3	5	14.0	4.4	18.6	74.8	3.7
C50S3R5	50	3	5	16.1	4.5	19.0	75.1	3.4
C75S3R5	75	3	5	17.0	4.6	19.2	75.4	3.2
C100S3R5	100	3	5	16.2	4.7	19.5	75.6	3.1
C0S4R5	0	4	5	15.8	4.2	18.4	74.8	3.7
C25S4R5	25	4	5	16.2	4.3	18.6	75.0	3.6
C50S4R5	50	4	5	16.8	4.4	18.9	75.2	3.5
C75S4R5	75	4	5	17.3	4.5	19.1	75.3	3.3
C100S4R5	100	4	5	16.5	4.6	19.3	75.5	3.2

**Table 7:** Comparative LCA Results.

Environmental Impact Category	Control Mix (0% CRMSP)	C75S3R5 Mix	C100S3R5 Mix
Global Warming Potential (kg CO_2_-eq)	100	92	88
Energy Consumption (MJ)	150	145	140

## Data Availability

Dataset available on request from the authors.

## References

[1] Li H., Han Y., Guangxun E., Sun Y., Wang L., Liu X., Ren J., Lin Z. (2024). Recycling of Waste Polyethylene in Asphalt and Its Performance Enhancement Methods: A Critical Literature Review. J. Clean. Prod..

[2] Jwaida Z., Dulaimi A., Bahrami A., Mydin M.A.O., Özkılıç Y.O., Jaya R.P., Wang Y. (2024). Analytical Review on Potential Use of Waste Engine Oil in Asphalt and Pavement Engineering. Case Stud. Constr. Mater..

[3] Hamid A., Baaj H., El-Hakim M. (2022). Rutting Behaviour of Geopolymer and Styrene Butadiene Styrene-Modified Asphalt Binder. Polymers.

[4] Uzun S., Terzi S. (2012). Evaluation of Andesite Waste as Mineral Filler in Asphaltic Concrete Mixture. Constr. Build. Mater..

[5] Choi M.J., Kim Y.J., Kim H.J., Lee J.J. (2020). Performance Evaluation of the Use of Tire-Derived Fuel Fly Ash as Mineral Filler in Hot Mix Asphalt Concrete. J. Traffic Transp. Eng. (Engl. Ed.).

[6] Yıldız K., Atakan M. (2020). Improving Microwave Healing Characteristic of Asphalt Concrete by Using Fly Ash as a Filler. Constr. Build. Mater..

[7] Gao H., An B., Lei X., Gao Y., Liu H. (2024). Utilization of Molybdenum Tailings as an Alternative Mineral Filler in Asphalt Mastic: Rheological Performance and Environmental Aspects. Case Stud. Constr. Mater..

[8] Huang J., Yan K., Wang M., Shi K., Li Y., Zhang Y. (2024). Performance Evaluation of SBS-Modified Asphalt Mixtures Incorporating Waste Tire Rubber and HDPE. Constr. Build. Mater..

[9] Tang N., Xue C., Hao G., Huang W., Zhu H., Li R. (2023). Sustainable Production of Eco-Friendly Rubberized Asphalt Binders through Chemically Crosslinking with Polymer Modifier. J. Clean. Prod..

[10] Aliakbari A., Hajikarimi P., Khodaii A. (2023). Investigation of the Effect of Acid Sludge Neutralization Treatment on Producing a Sustainable Eco-Friendly Additive for Bituminous Composites. Int. J. Pavement Eng..

[11] Yu X., Li D., Leng Z., Yao H., Wang S. (2023). Weathering Characteristics of Asphalt Modified by Hybrid of Micro-Nano Tire Rubber and SBS. Constr. Build. Mater..

[12] Cao Y., Li J., Liu Z., Li X., Zhang F., Shan B. (2022). Rheological Properties of Styrene-Butadiene-Styrene Asphalt Mastic Containing High Elastic Polymer and Snow Melting Salt. Polymers.

[13] Wang J., Li X., Sun G., Ma X., Du H. (2024). Experimental Investigation on the Performance of SBS-Modified Asphalt Waterproofing Membrane by Thermo-Oxidative Aging and Freeze–Thaw Cycle. Polymers.

[14] Zhu H., Xu G., Gong M., Yang J. (2017). Recycling Long-Term-Aged Asphalts Using Bio-Binder/Plasticizer-Based Rejuvenator. Constr. Build. Mater..

[15] Sadeghian M., Latifi Namin M., Goli H. (2019). Evaluation of the Fatigue Failure and Recovery of SMA Mixtures with Cellulose Fiber and with SBS Modifier. Constr. Build. Mater..

[16] Kim K.N., Le T.H.M. (2023). Durability of Polymer-Modified Asphalt Mixture with Wasted Tire Powder and Epoxy Resin under Tropical Climate Curing Conditions. Polymers.

[17] Duarte G.M., Faxina A.L. (2021). Asphalt Concrete Mixtures Modified with Polymeric Waste by the Wet and Dry Processes: A Literature Review. Constr. Build. Mater..

[18] (2008). Standard Test Method for Penetration of Bituminous Materials.

[19] (2006). Standard Test Method for Softening Point of Bitumen (Ring-and-Ball Apparatus).

[20] (2012). Standard Test Method for Viscosity Determination of Asphalt at Elevated Temperatures Using a Rotational Viscometer.

[21] (2020). Standard Test Method for Determining the Rheological Properties of Asphalt Binder Using a Dynamic Shear Rheometer.

[22] (2013). Viscosity Determination of Asphalt Binder Using Rotational Viscometer.

[23] (2010). Standard Test Method for Elastic Recovery of Bituminous Materials by Ductilometer.

[24] Emminger C., Cakmak U.D., Lackner M., Major Z. (2023). Mechanical Characterization of Asphalt Mixtures Based on Polymeric Resin and Thixotropic Filler as a Substitute for Bitumen. Coatings.

[25] (2004). Standard Test Method for Density, Relative Density (Specific Gravity), and Absorption of Coarse Aggregate.

[26] (2014). Standard Test Method for Sieve Analysis of Fine and Coarse Aggregates.

[27] (2004). Standard Test Method for Total Evaporable Moisture Content of Aggregate by Drying.

[28] (2021). Standard Test Method for Rapid Determination of Carbonate Content of Soils.

[29] (2017). Standard Test Method for Materials Finer than 75-Μm (No. 200) Sieve in Mineral Aggregates by Washing.

[30] (2008). Standard Test Method for PH of Aqueous Solutions with the Glass Electrode 1.

[31] (2008). Standard Test Methods for Density and Specific Gravity (Relative Density) of Plastics by Displacement.

[32] (1928). Test Method for Amount of Material Finer than No. 200 Sieve in Aggregates.

[33] (2015). Standard Specification for Flexible Cellular Materials—Sponge or Expanded Rubber 1, 2.

[34] (2004). Standard Test Method for Density, Relative Density (Specific Gravity), and Absorption of Fine Aggregate.

[35] (1989). Testing Aggregates. Method for Determination of Particle Shape Flakiness Index.

[36] (2014). Standard Test Method for Resistance to Degradation of Small-Size Coarse Aggregate by Abrasion and Impact in the Los Angeles Machine.

[37] (1997). Bulk Density (“Unit Weight”) and Voids in Aggregate.

[38] (1989). Test Method for Resistance of Plastic Flow of Bituminous Mixtures Using Marshall Apparatus.

[39] (2022). Standard Test Method for Effect of Moisture on Asphalt Mixtures.

[40] (2013). Standard Practice for Open-Graded Friction Course (OGFC) Mix Design.

[41] (2017). Standard Method of Test for Hamburg Wheel-Track Testing of Compacted Hot Mixtures.

[42] (2011). Standard Method of Test for Determining Dynamic Modulus of Hot-Mix Asphalt Concrete Mixtures.

[43] Zhang Q., Luo J., Yang Z., Wang J., Zhao Y., Zhang Y. (2023). Creep and Fatigue Properties of Asphalt Mastic with Steel Slag Powder Filler. Case Stud. Constr. Mater..

[44] Heijungs R., Guinée J.B., Huppes G., Lankreijer R.M., Udo de Haes H., Wegener Sleeswijk A., Ansems A.M.M., Eggels P.G., van Durin R., de Goede H.P. (1992). Environmental Life Cycle Assessment of Products: Guide and Backgrounds (Part 1). Environ. Sci..

